# Excitability properties of single human motor axons: are all axons identical?

**DOI:** 10.3389/fncel.2014.00085

**Published:** 2014-03-19

**Authors:** Lydia P. Kudina, Regina E. Andreeva

**Affiliations:** Institute for Information Transmission Problems of the Russian Academy of Sciences (Kharkevich Institute)Moscow, Russia

**Keywords:** motor control, voluntary muscle contraction, single firing motor units, axonal excitability recovery, superexcitability

## Introduction

Under natural motor control, repetitive firing of motoneurons is transmitted by their motor axons to muscle fibers in the “one to one” fashion that allows the analysis of human motoneuron firing via recordings of motor unit (MU) action potentials (see Kernell, 2006; Heckman and Enoka, 2012). However, when axons are diseased or damaged, local increasing axonal excitability may result in disturbing this basic principle and creating an additional focus of excitation in an axon itself, leading to different symptoms, including MU spontaneous discharges. In order to gain an insight into possible pathophysiological mechanisms underlying changes in axonal excitability, many studies were addressed exploring axonal excitability properties in healthy humans and their fundamental characteristics have been obtained (reviewed by Bostock et al., 1998; Burke et al., 2001; Nodera and Kaji, 2006). In particular, axonal activation has been found to be followed by certain excitability recovery cycle as tested commonly after a supramaximal conditioning stimulus (e.g., Kiernan et al., 1996; Bostock et al., 1998; Murray and Jankelowitz, 2011) or after the cessation of maximal voluntary muscle contractions (Vagg et al., 1998; Kuwabara et al., 2001; Rossi et al., 2012). Traditionally, axon excitability properties were explored for whole motoneuronal pool, using a compound muscle action potential (CMAP), without analysing the behavior of single MUs. However, such approach gives no information on the excitability properties of motor axons belonging to different types of MUs.

A few reports, beginning with the seminal studies of Bergmans (1970, 1973), addressed the excitability properties of single motor axons, as a rule, low-threshold to electrical stimulation and thus belonging to large MUs, high-threshold to voluntary muscle contraction (Borg, 1980; Bostock and Baker, 1988; Shefner et al., 1996; Hales et al., 2004; Bostock et al., 2005). At the same time there are no data on the excitability properties of axons belonging to small, slow MUs during their natural activation, while these MUs are essential part of motoneuronal pools as they are primarily activated during both voluntary movements and the maintenance of posture, as well as at reflex activation. The question arises of whether or not the excitability properties of these axons are similar to that of MUs with high-threshold to voluntary muscle contraction. Our findings reported below give some possibility to start the discussion of this question.

## Excitability properties of single motor axons belonging to slow MUs

Experiments were carried out on four healthy human volunteers, aged 46–62 years. The tibialis anterior (TA), flexor carpi ulnaris (FCU), and abductor pollicis brevis (APB) were investigated. The subject recruited a few MUs of a muscle under study by weak voluntary contraction and kept steady MU firing. It is commonly accepted that in normal motor behavior, MUs are typically used such that the most easily recruited, small MUs are the slowest ones (“size principle” of Henneman; for review see Henneman and Mendell, 1981; Kernell et al., 1999; Kernell, 2006). In our experiments, during gentle voluntary contraction the slowest MUs tend to be recruited. The potentials of single MUs were recorded using a bipolar needle electrode and stored on the magnetic tape for off-line analysis. Action potentials of each MU were identified on the basis of their amplitude and waveform shape. The results of the computer identification were verified by an experienced operator. Only the recordings of steady firing MUs with 100% proper identification were accepted for further analysis.

While the subject maintained muscle contraction, single (random in relation to MU background firing), low-intensity stimuli of 0.5–1.0 ms duration, at interstimulus interval of 1–5 s were applied through bipolar surface electrode to the following mixed nerves: common peroneal, ulnaris, or median nerve during TA, FCU, or APB studies, respectively. In response to weak stimulation of mixed nerve, low-threshold to voluntary contraction MUs commonly fire at the H-reflex latency. Their thin motor axons are normally high-threshold to electric stimulation and their selective activation is rather a challenge. However, in the majority of our experiments, we were successful in evoking M-response in some of the slow MUs, under conditions of a thorough manual adjustment of the stimulating electrode position.

For significant evaluation of MU responses to a test volley and their latencies, peri-stimulus time histograms (PSTHs) of single MUs were plotted. In order to estimate stimulation effect on regular motoneuron firing, for each MU, in each trial, a target interspike interval (ISI), in which the motor volley arrived, and a corresponding background ISI (just preceding an each stimulus) were calculated and their distributions for all trials were plotted.

Exploring excitability changes in an axon after propagation of a regular motoneuron discharge, we based on assuming that the most *functionally significant* measure of axon excitability recovery is axonal spike occurrence itself. Therefore, in each trial, the presence or absence of the M-response of an MU tested provided unequivocal evidence of whether or not axonal excitability was recovered. At multiple testing, changes in axonal excitability throughout the target ISI were estimated by the firing index (FI), showing the percentage of MU responses at the M-response latency to the total number of test volleys arriving in this step of a target ISI. Thus, the FI gave a quantitative measure of axonal excitability recovery after transmitting a regular voluntary discharge.

**Figure 1:**
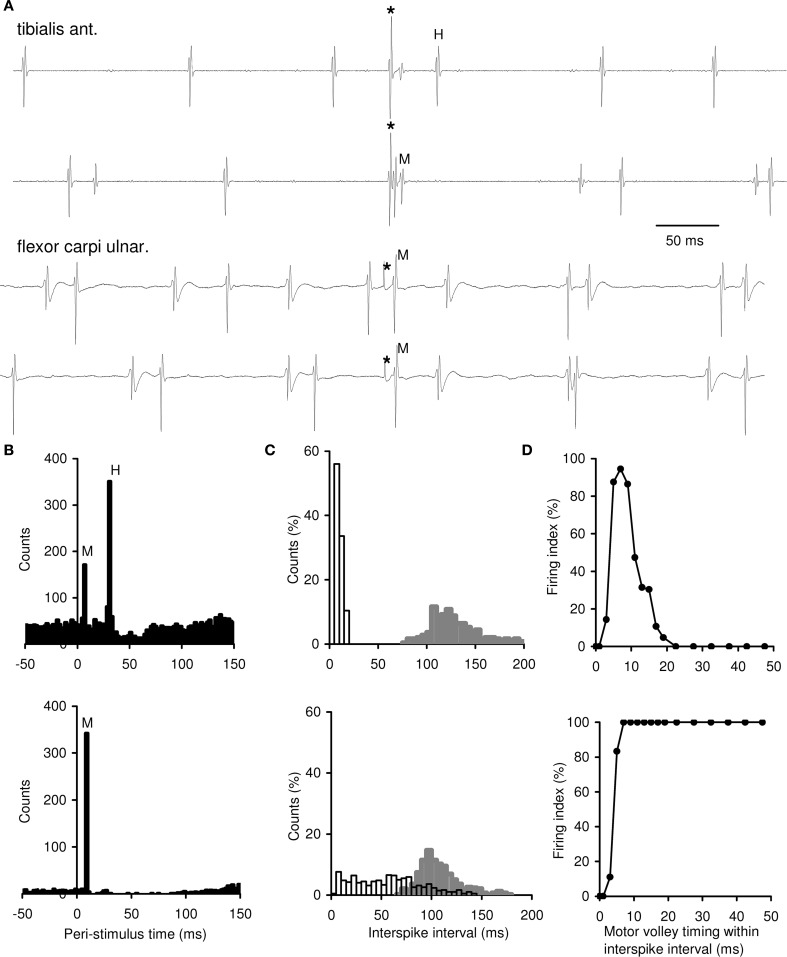
**Single motor axon excitability testing by mixed nerve stimulations during voluntary muscle contractions**. **(A)** Examples of MU recordings. Two top records, an MU of group 1 displaying both the H-reflex and M-response; two bottom records, an MU of group 2 displaying M-response only. Asterisks, stimulation time. **(B)** Peri-stimulus time histograms (PSTHs) of single MUs. Top, an MU of group 1 showing a significant increase in firing probability at the M-response and H-reflex latencies (the tibialis anterior, 2462 trials). Bottom, an MU of group 2 displaying a significant increase in firing probability at the M-response latency only (the flexor carpi ulnaris, 379 trials). M-response was observed in 6.9% trials in top PSTH and in 90.1% trials in bottom PSTH. Zero, stimulation time. The bin width, 2 ms. **(C)** Distributions of target and background ISIs of single MUs. Top, three MUs of group 1 from one experiment (the tibialis anterior); bottom, an MU of group 2 (the flexor carpi ulnaris). Target ISIs (open bars), *n* = 257 in top and *n* = 421 in bottom; background ISIs (filled bars) from the same trials. Bin width, 5 ms. **(D)** Testing of changes in axonal excitability throughout a target ISI of single MUs. Top, an MU of group 1 (the tibialis anterior, 897 trials); bottom, an MU of group 2 (the flexor carpi ulnaris, 294 trials). Changes in the firing index (FI) after a regular voluntary discharge (taken as 0 ms) are shown within the first 50 ms of the target ISI. The remaining part of the ISI, in which the FI continued to be equal to 0% (in top) and to 100% (in bottom), is not shown. Note bin width of 2 ms within 0–20 and 5 ms within 20–50 ms of the target ISI.

A total of 96 MUs were recorded (36 MUs of TA, 24 MUs of FCU, and 36 MUs of APB); 39 MUs (40.6%) exhibited M-responses and could be divided into two groups: MUs showing both M-responses and H-reflex (group 1) and those displaying M-responses only (group 2). Examples of MU recordings are presented in Figure 1A. Mean background firing rates of MUs in the muscles investigated ranged between 5 and 14 imp/s. The group 1 included 34 MUs that fired 430 responses to motor volleys. Some of these MUs exhibited very few M-responses; the others demonstrated the M-responses more frequently (25–154, mean 54.2 responses per an MU). For these MUs, PSTHs revealed a significant increase in MU discharge probability at both the M-response and H-reflex latencies (Figure 1B, top). The MUs from group 2 displayed a significant increase in MU firing probability at the M-response latency alone (Figure 1B, bottom). This type of behavior was encountered only in 5 MUs (4 MUs in FCU and 1 MU in APB) from 3 experiments in two out of four subjects. However, in each of the MUs of group 2, the motor axon stimulation elicited M-responses in the most trials, in contrast to the MUs of group 1. The target ISIs for MUs of both groups were significantly shortened as compared with background ones (see Figure 1C).

To understand the mechanisms underlying different character of MU responses to motor volley, we explored the axonal excitability recovery after a regular discharge for MUs of group 1 and group 2 (Figure 1D, top and bottom, respectively). When the motor volley arrived just in the beginning of the target ISI, it did not evoke an M-response in the both MU groups, and FI was equal to 0% (the refractory period). About 3–5 ms after a regular discharge, as refractoriness subsided, the MUs began to respond to a motor volley and FI sharply rose. At 5–8 ms of target ISI, FI reached to 90–100%. Further, for MUs of group 1, FI fell to zero at 12–19 ms; thereafter, the motor volley appeared ineffective again up to the end of an ISI (FI = 0%). In contrast, MUs of group 2, responded to a motor volley at *any moment* of the target ISI, with FI up to 100%. Thus, axons belonging to MUs of group 1, after transmitting a regular discharge, displayed early and late irresponsive periods and a *short-lasting* period of high responsiveness to the motor volley. MUs of group 2 were characterized by a *long-lasting* period of axonal responsiveness with no late irresponsiveness.

## Discussion

The present study provides data on excitability changes in single slow motor axons (belonging to small MUs) transmitting natural motoneuron firing during gentle voluntary muscle contractions. To our knowledge, the question has not been studied before. The axonal excitability recovery of the majority of small MUs was found to be qualitatively similar to that reported previously for both CMAP and single large MUs with a high threshold for voluntary muscle contractions.

However, apart from MUs with usual axonal excitability recovery, in two out of four healthy subjects, we had the opportunity to record some MUs displaying the especial excitability properties in axons of the ulnaris and median nerves. It has been revealed that after the short irresponsive period, these MUs were able to respond to motor volley within an ISI throughout. The long duration of the responsibility could be surprising, but it is consistent with data by Bergmans (1970), who pioneered the study of single human motor axons by surface stimulation. He has reported that two axons of the median nerve were found to have the unusual excitability recovery cycle: a long duration supernormal period with no late subnormality. In our experiments, four out of five MUs with especial axonal excitability properties were recorded in FCU supplied by the ulnaris nerve. However, at present, there are no sufficient data to discuss, in total, the possible differences in axonal excitability properties among the muscles investigated.

Hence, the population of small MUs was found not to be homogenous as their motor axons were not identical and displayed different excitability properties after the transmission of a regular motoneuron discharge. Previously, Kiernan et al. (1996), analysing the axonal recovery cycle after a supra-maximal conditioning stimulus with using test stimulus evoking CMAP of 30, 50, and 70% max, have concluded that there is no difference in excitability recovery of axons with different thresholds. Obviously, MUs with thin axons (like those from the present experiments) did not contribute to the CMAPs above. It is important to note that using CMAP can hardly give any possibility to analyse excitability recovery in small, slow MUs because they are commonly “dissembled” in the CMAP, whose the main characteristics, such as the latency and amplitude, are generally controlled by large MUs with fast axons. Therefore, we are of opinion that it is necessary further investigations of excitability properties of slow motor axons using single MU recordings but not only CMAP.

The next question that arises is: what is mechanism underlying this unusual excitability property revealed in some motor axons? It is widely accepted that the depolarizing after-potential underlies the super-excitability phase lasting up to some 20 ms in the recovery cycle of large axons (see Burke et al., 2001). The same mechanism obviously underlies the short responsive phase in the axonal recovery cycle of small MUs of group 1 from our experiments. At the same time, our findings on a prolonged, broad responsible period in axonal excitability recovery with no irresponsiveness throughout the whole ISI revealed in the MUs of group 2 are not readily explained based on this mechanism alone. What are additional mechanisms that could conceivably contribute to the phenomenon? Bostock et al. (1991) have provided evidence that fast motor axons can display two stable states (high- and low-threshold) following ischemia. In the study of Bostock and Bergmans (1994), it has been suggested that post-tetanic ectopic discharges in motor axons depended on the bistability of the axonal membrane potential and “occur on transitions from a hyperpolarized to a depolarized state. The transitions may occur spontaneously, but are readily triggered by an action potential, giving rise to a prolonged supernormal period.” Following these suggestions, it might be proposed that MUs with slow axons could presumably possess the similar ability for two threshold states and that transition to a low-threshold state resulted in a prolonged responsive phase in the excitability recovery cycle (distinctive axonal plateau potential?). The further exploration is required to clarify the underlying mechanisms.

## Conclusion

In normal motor behavior, an axon is only a transmitter of motoneuron firing without any discharge distortion. If so, within a given MU, the excitability recovery cycle in the axon has to be some counterpart of that in the motoneuron, certainly including late irresponsive period (equivalent of the after-hyperpolarization which inevitably follows each spike of *each* motoneuron). However, axons of low-threshold MUs were found not to be identical as some of them, after refractoriness, displayed prolonged responsiveness with no late irresponsible period. In this case, the axonal capacity for transmitting spikes without late irresponsiveness must be unclaimed. An important question is obviously: what are the benefits of such axonal property in normal motor control? This is presently no answer to this question. On the other hand, in neuromuscular diseases, it may be assumed that axons with a long-lasting responsive period can be predisposed to dysfunction to a greater extent than others, in particular, to creating an additional focus of excitation in an axon itself, leading to MU spontaneous firing that emphasizes the importance of further analysis of the excitability properties of similar axons in healthy subjects.
